# The Therapy of SARS-CoV-2 Infection in Children

**DOI:** 10.3390/jcm13010120

**Published:** 2023-12-25

**Authors:** Kathryn M. Edwards

**Affiliations:** Division of Infectious Diseases, Department of Pediatrics, Vanderbilt University School of Medicine, Nashville, TN 37232, USA; kathryn.edwards@vumc.org; Tel.: +1-615-322-2250

**Keywords:** COVID-19, COVID-19 drug treatment, pediatrics

## Abstract

The impact of SARS-CoV-2 infections in children has fortunately been lower than what has been seen in adults. However, even previously healthy children have developed severe disease, sometimes with subsequent mortality, and those who are infants or adolescents, are from racial and ethnic minority groups, or have certain chronic conditions are at higher risk of these outcomes. During the pandemic, extensive studies of therapeutic agents, including antivirals and immunomodulators, were conducted in adults. Few trials included children, and most were in older children and adolescents. Thus, the potential benefits of therapies in children must be extrapolated from adult evidence. Despite these limitations, advisory committees of the National Institute of Health (NIH), the Infectious Disease Society of America (IDSA), and the Pediatric Infectious Diseases Society (PIDS) were constituted, and expert consensus guidelines were developed. This review provides a synthesis of those comprehensive recommendations for therapy in children. These address treatment during the early infectious period with antiviral agents, including remdesivir and nirmatrelvir/ritonavir, as well as treatment in the later period of immune dysregulation with corticosteroids and immunomodulators. In addition, the therapeutic approach for multisystem inflammatory syndrome in children (MIS-C), also referred to as Pediatric Inflammatory Multisystem Syndrome temporally associated with SARS-CoV-2 (PIMS-TS), is also provided.

## 1. Introduction

The management of SARS-CoV-2 infections in children is our focus. Fortunately, the impact of this infection on children has been lower than what has been seen in adults, particularly in older individuals [1,2]. However, multiple risk factors have been associated with greater morbidity and mortality in children [3,4,5,6,7,8], including cardiac disease, neurologic disorders, prematurity, diabetes, obesity (particularly severe obesity), chronic lung disease, feeding tube dependence, and immunocompromised status. Age (<1 year and 10–14 years) and being from a racial and ethnic minority group have also been associated with severe disease.

During the SARS-CoV-2 pandemic, studies of therapeutic agents, including antivirals and immunomodulators, were primarily undertaken in adults, with few trials including children. Most children who were included in the therapeutic trials were older children or adolescents, with few studies devoted to young children, particularly premature infants and newborns. As the epidemic subsided, the pediatric trials were able to enroll few participants, yielding limited results as to the safety and efficacy of the interventions evaluated. As a result, potential benefits of therapies in children must be extrapolated from adult evidence. Despite these limitations, advisory committees of the National Institute of Health (NIH), the Infectious Disease Society of America (IDSA), and the Pediatric Infectious Diseases Society (PIDS) were constituted, and their literature review and expert consensus has contributed helpful information for the management of children with SARS-CoV2 infection. These expert consensus guidelines will continue to be updated as additional data become available. This review will be a synthesis of those comprehensive recommendations; the reader is referred to them for additional details [9,10,11,12].

The pathogenesis of COVID-19 and its ultimate management can be divided into two phases. The early phase of the infection is mainly involved with the growth of the SARS-CoV-2 virus and can be best addressed through the administration of antiviral agents. Later in the clinical course, a dysregulated immune/inflammatory response is the hallmark, and this phase is best addressed with immunomodulating therapies. Each of these phases and their associated therapies will be discussed. I will also briefly address the use of monoclonal antibodies and the treatment of multisystem inflammatory syndrome in children. Again, it should be stressed that the data on therapies used in children are often dependent upon extrapolation of the data accumulated in adults, and the treatment of infants and young children is even more complicated by restrictions on use of many of the therapeutic agents to older children.

## 2. Antiviral Agents

Antiviral agents are important in the early stages of infection, and their prompt initiation improves the prognosis in many situations, as outlined below. Three antiviral agents evaluated during the SARS-CoV-2 pandemic were found to be therapeutically effective: remdesivir, nirmatrelvir/ritonavir, and molnupiravir. Each agent is summarized in Table 1 and discussed below.

Remdesivir: Remdesivir is an antiviral agent that triggers the premature termination of viral RNA transcription and has in vitro activity against both MERS-CoV and SARS-CoV-1 and -2 [14,15]. Several randomized clinical trials (RCTs) in adults, conducted early in the pandemic, compared remdesivir to a placebo and found the drug to be safe and effective [16,17,18,19]. Small outpatient studies in children with COVID-19 who were at risk for severe disease found the drug to be well tolerated and effective, but there have been no RCTs assessing the efficacy of remdesivir in hospitalized pediatric patients. Compassionate use studies in children confirmed its tolerability and low rate of serious adverse events [20] and established the dose of 5 mg/kg on day one (maximum dose 200 mg) followed by 2.5 mg/kg daily. The US Food and Drug Administration (FDA) has approved remdesivir for children ≥ 28 days of age who weigh ≥ 3 kg. Currently, a three-day course of remdesivir is recommended for children with mild-to-moderate COVID disease who are at high risk for severe COVID-19. Remdesivir therapy for up to five days is also recommended for severe COVID-19 in hospitalized patients with SpO_2_ ≤ 94% on room air. It is, however, not recommended for patients on invasive ventilation and/or extracorporeal membrane oxygenation (ECMO). One major limitation is that it must be administered by the intravenous route.

Nirmatrelvir/ritonavir: Nirmatrelvir, a substrate of the cytochrome P450 3A4 isoenzyme system, is an inhibitor of the principal protease of SARS-CoV-2 and blocks viral replication. It is co-packaged with ritonavir, a potent inhibitor of cytochrome P450 3A4, resulting in higher drug concentrations and a longer half-life, facilitating oral dosing every 12 h [21]. One large RCT compared ritonavir-boosted nirmatrelvir with a placebo in adults who were at high risk for severe COVID-19 and showed an 89% reduction in COVID-19-related hospitalization or all-cause mortality [22]. However, no pediatric patients were included in the trial. Since ritonavir has been used extensively in HIV and hepatitis C management in children, several lines of evidence support the recommendation for the use of ritonavir-boosted nirmatrelvir for the treatment of mild-to-moderate COVID-19 in adolescents at risk for severe COVID-19. These include the proven efficacy of ritonavir-boosted nirmatrelvir in adults, its acceptable safety profile, the previous pediatric clinical experience with ritonavir, and the availability of an oral formulation. Nirmatrelvir/ritonavir is only approved by the FDA in patients ≥ 18 years old, but an Emergency Use Authorization (EUA) exists for high-risk patients ≥ 12 years old who weigh ≥ 40 kg [21]. Using nirmatrelvir/ritonavir early in the disease course, within five days of symptom onset, confers maximum benefit. Other medications that the patient may be receiving must be screened for drug interactions with this combination antiviral [23]. Guidance on managing these drug interactions can be found on several websites [23,24,25,26]. Recurrence of symptoms after stopping nirmatrelvir/ritonavir occurs in 0.8% to 6.6% of patients [27,28,29]. However, repeat nirmatrelvir/ritonavir treatment is not recommended.

Molnupiravir: Molnupiravir is an oral antiviral that is incorporated into the viral RNA, leading to serial mutations and inhibition of viral replication [30,31]. Early phase 1 trials in adults reported the drug to be well tolerated, without serious adverse events [32], and the drug was granted an EUA at the end of 2021. However, it is only recommended for treatment in nonpregnant adults given the concerns for mutagenesis, and it is not recommended in children because it has been shown to interfere with bone and cartilage formation in experimental animals [33]. Since the EUA was issued, some reports have implied that molnupiravir has a lower clinical efficacy than the two other treatment options [34]. No randomized clinical trials have compared the agents head-to-head.

## 3. Immunomodulatory Agents

Immunomodulatory agents are important in combating the second phase of the infection that is characterized by immune system dysregulation. Again, most of the studies of these agents have been conducted in adults, with few children included in the trials, and most that were included were adolescents. The assessment of these agents in adults and their extrapolation to adolescents and children will be outlined below.

Corticosteroids: Early in the SARS-CoV-2 pandemic, systemic corticosteroids were not recommended because of concerns for worsening clinical status, delayed viral clearance, and superimposed infections [35,36,37]. However, given the hyper-inflammatory state in COVID-19, steroids, particularly dexamethasone, and other immunomodulatory approaches were rapidly evaluated. Although many clinical trials with corticosteroids were performed, one of the largest and most impactful was the RECOVERY trial, a randomized trial among critically ill hospitalized adults in the United Kingdom [38]. Mortality was 34% less among patients treated with dexamethasone than those not treated. A systematic review of multiple additional studies of corticosteroid use supported the safety of this approach [39]. RCTs of corticosteroid use in hospitalized children were not conducted. However, the results of the RECOVERY trial demonstrating the significant reduction in mortality with the use of corticosteroids in adults support the recommendation for their use in children who require mechanical ventilation or ECMO and those who require oxygen delivered by a high-flow device or noninvasive ventilation (NIV). Finally, routine corticosteroid use is not recommended for children solely on conventional oxygen, but it should be considered with remdesivir in children, particularly adolescents, with increasing oxygen needs. There are no data to support its use in non-hospitalized patients with COVID-19 and it is not recommended in pediatric outpatients with COVID-19.

Inhaled steroids: Several randomized clinical trials evaluated the use of inhaled corticosteroids for ambulatory or hospitalized adult patients with mild-to-moderate COVID-19 [40,41,42,43]. Inhaled corticosteroids did not demonstrate a beneficial effect on mortality or hospitalization. Thus, guideline panels suggest against inhaled corticosteroids for the treatment of patients with mild-to-moderate COVID-19. Patients already on inhaled corticosteroids may continue them.

Several immunomodulators other than corticosteroids have been recommended for use in children with severe COVID-19 disease; they can be grouped into two therapeutic groups. The mechanism of action of one group of these agents is inhibition of Janus kinase (JAK) with subsequent “suppression of the signal transducer and activator of transcription (STAT) proteins leading to reduced inflammatory mediators” [44]. The second group of immunomodulators inhibit interleukin-6 (Il-6), a proinflammatory cytokine produced by lymphocytes, monocytes, and fibroblasts. Infection by SARS-CoV triggers production of IL-6, which promotes systemic inflammation and hypoxemic respiratory failure [45].

Janus kinase inhibitors: Several JAK inhibitors are available but only baricitinib and tofacitinib have been studied for COVID-19 treatment.

Baricitinib: Several large adult randomized clinical trials showed that some patients who needed additional supplemental oxygen and most patients who needed oxygen delivered through mechanical ventilation, NIV, or a high-flow device benefited from both corticosteroid use and a JAK inhibitor [46,47,48]. The RECOVERY trial included 33 children aged 2 to 17 years and showed that baricitinib conferred a survival benefit in hospitalized patients, with the greatest benefit in patients requiring oxygen supplementation through a high-flow device or NIV. Other trials supported its overall benefit and safety in patients with COVID-19. Although no studies have been performed primarily in children, earlier open-label trials and cohort studies of baricitinib in children as young as <5 years with rheumatic and autoinflammatory diseases demonstrated its safety. Unfortunately, the pharmacokinetics of baricitinib in younger children are poorly studied [49,50]. Its assessment in children with COVID-19 is restricted to only case reports.

The FDA approved the use of baricitinib in hospitalized adults with COVID-19 requiring supplemental oxygen, NIV, mechanical ventilation, or ECMO. An EUA was also issued for baricitinib treatment in hospitalized children requiring supplemental oxygen, NIV, mechanical ventilation, or ECMO between the ages of 2 and 17. 

Generalizing from the RCT in adults, baricitinib should be considered for children who require oxygen through a high-flow device, NIV, mechanical ventilation, or ECMO and whose oxygenation is not rapidly improved within the first 24 h on corticosteroids.

Tofacitinib: Results of clinical trials with tofacitinib conducted in hospitalized adults with COVID-19 pneumonia showed a decline in mortality and respiratory failure at Day 28 [51]. However, tofacitinib is not as well studied as baricitinib in adults with COVID-19, and remains an alternative. Although no clinical trials were conducted on tofacitinib in children with COVID-19, there is greater clinical experience with its use than baricitinib for juvenile idiopathic arthritis (JIA) in children as young as 2 years of age [52]. Phase 1 studies of tofacitinib in children established the pharmacokinetics and safety and a phase 3 RCT in children with JIA also demonstrated its safety [53]. Tofacitinib is available in a liquid formulation. Given its established safety in children, tofacitinib should be regarded as an alternative for children hospitalized for COVID-19 if baricitinib is not available. Although the dose of tofacitinib to treat hospitalized children with COVID-19 has not been established, it should likely be higher than the dose used to treat pediatric rheumatologic diseases.

Inhibition of interleukin-6: Two anti–IL-6 receptor monoclonal antibodies (mAbs), tocilizumab and sarilumab, were both studied in hospitalized patients with COVID-19. In late 2022, the FDA approved the intravenous use of tocilizumab for the treatment of COVID-19 in hospitalized adults who are receiving systemic corticosteroids and require supplemental oxygen, NIV, mechanical ventilation, or ECMO [54].

Tocilizumab: Two large RCTs in hospitalized adults with COVID-19 showed that mortality was reduced with tocilizumab [55,56]. In the RECOVERY trial, hospitalized adults with an oxygen saturation of <92% on room air or who were receiving supplemental oxygen therapy and had C-reactive protein levels of ≥75 mg/L were randomized to either tocilizumab or a placebo [55]. At 28 days, the mortality was significantly lower in the tocilizumab arm. The REMAP-CAP trial randomized adults with suspected or confirmed COVID-19 admitted to an intensive care unit who were receiving either respiratory or cardiovascular support to either tocilizumab, sarilumab or a placebo [56]. The mortality was reduced in the combined tocilizumab or sarilumab arm when compared to the placebo. Only case series have reported the treatment of severe pediatric COVID-19 with tocilizumab [57,58,59]. However, studies in non-COVID-19 pediatric conditions, including juvenile idiopathic arthritis and chimeric antigen receptor T cell-related cytokine release syndrome, have been conducted [57,58,59], and tocilizumab is FDA-approved for these indications. Inferring from the results of clinical trials among adults with COVID-19, children aged 2 to 17 years who require oxygen through a high-flow device, NIV, mechanical ventilation, or ECMO and who do not have rapid (e.g., within 24 h) improvement in oxygenation with corticosteroids should be considered for tocilizumab therapy.

Sarilumab: Data evaluating the efficacy of sarilumab for the treatment of COVID-19 hyperinflammation in children are limited, with no pediatric dosing information. Thus, sarilumab use in hospitalized children with COVID-19, except in a clinical trial, is not recommended.

## 4. Anticoagulation in Children with COVID-19

Coagulopathy is a prominent feature of severe COVID-19 in adults. However, there are limited data to determine the risk of thromboembolic disease in children with COVID-19 [60,61,62,63]. In one multicenter, retrospective study that included 814 pediatric patients, thromboembolic events were detected in 2.1% of patients with COVID-19 and in 6.5% of patients with MIS-C [62]. In the COVAC-TP trial, which evaluated anticoagulant prophylaxis in children hospitalized with COVID-19 or MIS-C, thromboembolic events occurred in only two patients [63]. Given these data, there is insufficient evidence to recommend either for or against the use of therapeutic anticoagulation in children of any age with COVID-19.

## 5. Monoclonal Antibodies

Early in the pandemic, neutralizing monoclonal antibodies directed against the spike protein of SARS-CoV-2 and pooled antibody preparations were used for both pre- and post-exposure prophylaxis and treatment. The emergence of variant viruses has left no antibody products that neutralize the circulating viruses in the U.S. Pediatric-specific data are limited or lacking for all neutralizing monoclonal antibody products, but suggest that the risk/benefit ratios for the use of SARS-CoV-2 monoclonal antibodies are likely similar between children and adults [64].

## 6. Multisystem Inflammatory Syndrome in Children

Multisystem inflammatory syndrome in children (MIS-C), also called Pediatric Inflammatory Multisystem Syndrome temporally associated with SARS-CoV-2 (PIMS-TS), is a rare acute inflammatory syndrome reported in children in the weeks following acute SARS-CoV-2 infection. Case definitions for this syndrome were derived from reports of critically ill children presenting with fever, rash, conjunctivitis, abdominal complaints, shock, and significant cardiac dysfunction in the setting of a recent SARS-CoV-2 infection [65,66,67,68,69,70,71,72,73,74,75,76,77]. The condition was most common among children between 6 and 10 years of age. Epidemiologic data showed clusters of MIS-C cases within 2–5 weeks following peaks of SARS-CoV-2 circulation and supported that it resulted from a delayed immunologic response.

Once the diagnosis of MIS-C is made, immunomodulatory medications are the mainstay of therapy. Intravenous immunoglobulin (IVIG) and systemic corticosteroids are frequent initial therapeutic choices [74,78]. Studies comparing outcomes after initial treatment using IVIG alone, steroids alone, or a combination of IVIG and steroids have led to differing conclusions, but the combination of both has been reported to lead to a faster and more sustained resolution of fever than IVIG alone [79]. Biologic treatments, including anakinra, infliximab, or tocilizumab, have also been used in refractory cases [80], though data are limited to inform the choice among these interventions or to infer those patients who would benefit most. Despite these limitations, overall outcomes in children with MIS-C have been generally good with few fatalities reported. In addition, with the evolution of the virus and individual immunologic experience with the virus by immunization or natural infection, the rates of MIS-C have markedly declined.

## 7. Conclusions

As has been stressed, most of the therapeutic trials conducted in patients with COVID-19 have been in adults, with few trials enrolling children. In those trials with pediatric participants, most have been older children or adolescents. However, given these limitations, I have tried to provide a brief overview of the complex therapeutic management of children with SARS-CoV-2 infection. These recommendations, which derive from those of the NIH, IDSA, and PIDS, must be applied in the context of the individual patient and are only suggestions for use in such patients. The more comprehensive data on the websites of these organizations provide additional rationale and discussion. They will also be regularly updated to contain any new results from clinical trials and retrospective data analyses. The applicability of earlier studies may decrease with continued evolution of factors that affect pediatric COVID-19 prevalence and severity. These include shifts in infection-associated immunity, vaccine uptake, and use of non-pharmacologic prevention measures, as well as the emergence of new SARS-CoV-2 variants with resulting implications for vaccine and therapeutic efficacy. Given the limitations of the data available, I have provided the rationale for when therapeutic agents are recommended and when they are not. As the pandemic wanes and both vaccine-induced and natural immunity occur in children, it is hoped that fewer patients will develop severe or critical illness and need these therapeutic interventions.

## Figures and Tables

**Table 1:** COVID-19 antivirals and pediatric use [13].

Antiviral	Timing	Age and Weight	Route and Course	Hepatic Considerations	Renal Considerations	Major Concerns
Remdesivir (Veklury™)	≤7d of symptoms	≥28 d and ≥3 kg	Intravenous daily for 3 days	Causes mild to moderate transaminase increases	None	Logistics
Nirmatrelvir/ ritonavir (Paxlovid™)	≤5 d of symptoms	≥12 y and ≥40 kg	Oral twice daily for 5 days	Not recommended if Child–Pugh class C disease	GFR 30–59: use 150 mg nirmatrelvir (50% dose reduction) GFR < 30: not recommended	Drug–drug interactions
Molnupiravir (Lagevrio™)	≤5 d of symptoms	≥18 y and ≥40 kg	Oral twice daily for 5 days	None	None	Mutagenicity/ teratogenicity prevent pediatric use; lower effectiveness
